# The Color of Water

**Published:** 1885-01

**Authors:** 


### THE COLOR OF WATER.

It is well known that the water of different lakes and rivers differs in color. The Mediterranean Sea is indigo blue, the ocean sky blue, Lake Geneva is azure, while the Lake of the Four Forest Cantons and Lake Constance, in Switzerland, as well as the river Rhine, are chrome green, and Klœnthaler Lake is grass green.

Tyndall thought that the blue color of water had a similar cause as the blue color of the air, being blue by reflected light and red by transmitted light. M. Spring has recently communicated to the Belgian Academy the results of his investigations upon the color of water. He proved that perfectly pure water in a tube 10 meters long had a distinctly blue color, while it ought, according to Tyndall, to look red. Spring also showed that water in which carbonate of lime, silica, clay, and salts were suspended in a fine state of division offered a resistance to the passage of light that was not inconsiderable. Since the red and violet light of the spectrum are much more feeble than the yellow, the former will be completely absorbed, while the latter passes through, producing, with the blue of the water itself, different shades of green.

